# Randomized controlled phase I/II study to investigate immune stimulatory effects by low dose radiotherapy in primarily operable pancreatic cancer

**DOI:** 10.1186/1471-2407-11-134

**Published:** 2011-04-13

**Authors:** Carmen Timke, Hubertus Schmitz Winnenthal, Felix Klug, Falk FF Roeder, Andreas Bonertz, Christoph Reissfelder, Nathalie Rochet, Moritz Koch, Christine Tjaden, Markus W Buechler, Juergen Debus, Jens Werner, Philipp Beckhove, Jürgen Weitz, Peter E Huber

**Affiliations:** 1 Department of Radiation Oncology, German Cancer Research Center and University Hospital Center, Heidelberg, Germany; 2 Department of General, Visceral and Transplantation Surgery, University Hospital Center Heidelberg, Germany; 3 Translational Immunology Unit, German Cancer Research Center, Heidelberg, Germany

**Keywords:** pancreatic cancer, immune therapy, low dose radiation, T-cells

## Abstract

**Background:**

The efficiencies of T cell based immunotherapies are affected by insufficient migration and activation of tumor specific effector T cells in the tumor. Accumulating evidence exists on the ability of ionizing radiation to modify the tumor microenvironment and generate inflammation. The aim of this phase I/II clinical trial is to evaluate whether low dose single fraction radiotherapy can improve T cell associated antitumor immune response in patients with pancreatic cancer.

**Methods/Design:**

This trial has been designed as an investigator initiated; prospective randomised, 4-armed, controlled Phase I/II trial. Patients who are candidates for resection of pancreatic cancer will be randomized into 4 arms. A total of 40 patients will be enrolled. The patients receive 0 Gy, 0.5 Gy, 2 Gy or 5 Gy radiation precisely targeted to their pancreatic carcinoma. Radiation will be delivered by external beam radiotherapy using a 6 MV Linac with IMRT technique 48 h prior to the surgical resection. The primary objective is the determination of an active local external beam radiation dose, leading to tumor infiltrating T cells as a surrogate parameter for antitumor activity. Secondary objectives include local tumor control and recurrence patterns, survival, radiogenic treatment toxicity and postoperative morbidity and mortality, as well as quality of life. Further, frequencies of tumor reactive T cells in blood and bone marrow as well as whole blood cell transcriptomics and plasma-proteomics will be correlated with clinical outcome. An interim analysis will be performed after the enrolment of 20 patients for safety reasons. The evaluation of the primary endpoint will start four weeks after the last patient's enrolment.

**Discussion:**

This trial will answer the question whether a low dose radiotherapy localized to the pancreatic tumor only can increase the number of tumor infiltrating T cells and thus potentially enhance the antitumor immune response. The study will also investigate the prognostic and predictive value of radiation-induced T cell activity along with transcriptomic and proteomic data with respect to clinical outcome.

**Trial registration:**

ClinicalTrials.gov - NCT01027221

## Background

Pancreatic cancer is still one of the most lethal cancers with very unfortunate prognosis. The mortality rate follows closely the incidence rate. It ranks 9^th ^in incidence but 4^th ^in cancer related death [1], leaving the patients at first diagnosis with an overall median survival of 6-10 month. So far, surgery is the only chance of cure in this devastating disease [2]. However, intended curative surgery is possible in about 20% only and even these patients have a median survival expectancy of less than two years [3].

Unfortunately, many treatment studies including surgery, chemotherapy and conventional radiotherapy have shown little progress in improving the prognosis of pancreatic cancer patients in the last decades. Therefore, novel alternatives such as therapies that alter the immune response are of high interest in this type of cancer. This is even more the case, since in pancreatic cancer several ways warding off spontaneously induced immune responses have been described [4]: The aggressive nature of pancreatic ductal carcinoma is partially due to its tumor microenvironment with stellate cells producing the typical excessive desmoplasia [5,6]. It has been shown in experimental cancer models in nude mice that tumors grew faster when pancreatic cancer cells and stellate cells were injected together [7]. Stellate cells can support growth, invasion, metastasis and chemoresistance of tumors. Furthermore through recruitment and activation of stroma cell populations, pancreatic cancers generate a predominantly immune -suppressive microenvironment [8]

T-cell responses are partially regulated by dendritic cells, which take up antigens. Dendritic cells present the antigens to naïve T-cells and thereby activate them. Cytokines like type 1 interferon and extracellular matrix degregation products enhance dendritic cell activation, while IL-10 and TGF-β1 inhibit dendritic cell activation. In pancreatic cancer IL-10 and TGF-β1 are produced by stellate cells, cancer infiltrating macrophages and mast cells or regulatory T-cells [9,10]. IL-10 and TGF-β1 can also be upregulated by irradiation e.g. in endothelial cells [11].

In pancreatic cancer MHC molecules and the Fas-receptor are downregulated making the cells more resistant to recognition and lysis by activated T-cells. Conversely, ionizing radiation has been shown to upregulate MHC molecules and Fas-receptors in vivo [12].

Gastrointestinal tumours, including pancreatic cancer induce, attract and maintain regulatory T-cells, which inhibit effector T-cells activation and function [13,14]. Regulatory T cells and effector T-cells exhibit differences in their radiosensitivity [15] which opens a dose window for radiation induced immunotherapy.

Radiotherapy is a substantial part in the multimodal treatment of many solid cancers. The general understanding of the biology of radiation has been dominated by mitotic-catastrophic or apoptotic cell death. In addition to the classical effects such as DNA damage radiation also affects most other cell signaling circuits including MiRNA [16] which affects both the tumor cell and normal cell compartments with all aspects of the tumor microenvironment [17]. Although radiation treatment has been known to be immunosuppressive, there is emerging evidence, that the effectiveness of radiation therapy can in part be due to its immunostimulating consequences [18,19].

Potential mechanisms leading to immune response for ionizing radiation involve activation of stress response pathways e.g. activation of p53 and NF-κB. These abd others can regulate expression of molecules that promote a proinflammatory immune response including TNF-α, Interleukin-1 (IL-1), ICAM-1, VCAM-1, MHC molecules or PDGF [20]. Stimulation of the NF-κB and the direct cell death resulting from ionizing radiation also stimulates invasion and activation of leukocytes leading to a productive immune response [21].

Cytokines can act to inhibit immune responses, e.g. IL-10 and TGF-β, while others like TNF-α, IFN--α and IL-1 are proinflammatory [22]. TGF-α and IL-1 have been found to increase after sublethal total body irradiation, as have TGF-β and IL-6 [23]. The release of these cytokines leads to recruitment and activation of leukocytes from peripheral blood and extravasation into tissue and tumors.

Insufficient migration of effector T lymphocytes may considerably account for the hitherto low clinical efficiency of clinical immunotherapy. Adhesion molecules such as ICAM-1, E- and particularly P-Selectin are located on the endothelial cell surface and mediate the immigration of effector T lymphocytes and other immune cells into human epithelial tumours [24]. Vascular endothelial cells upregulate ICAM-1 and E-Selectin as a response to ionizing radiation and thereby facilitate leukocyte arrest and transmigration [25].

Chemokines are chemotactic cytokines that facilitate directional migration of cells expressing a cognate chemokine receptor. Two important chemokines regulated by ionizing radiation are CXCL-16 and SDF-1 [26]. Mice deficient of CXCR6 (the CXCL-16 responding receptor) have been demonstrated to exhibit decreased CD8+ cell recruitment and radiotherapy responsiveness [27]. It has also been demonstrated, that macrophage infiltration following RT was dependent on the SDF-1α expression. Thus ionizing radiation can regulate chemokines either via recruitment of tumor suppressive CD8 cells or tumor promoting cells such as macrophages.

Functional antigen-presentation is required for a productive anti-tumor T-cell response. Antigen-Presenting cells capture antigens and following processing present those on their cell surface vial major histocompatability complexes (MHC). T-cells recognize antigens bound to MHC and initiate anti-tumor response. Ionizing radiation also upregulates MHC class I on tumor cells and antigen-presenting cells [28] indicating that ionizing radiation can enhance tumor cell recognition by T-cells.

Moreover, ionizing radiation can activate endothelial cells thus facilitating the immigration of T-cells into the tumor. Overall, the available preclinical in vivo data support the hypothesis that radiation may provide at least some of the necessary maturation signals.

To date, no systematic clinical trial on the question if and how low dose radiotherapy can enhance immune activity in tumors. One question to address this is to determine the effective radiation dose to cause immune response. Furthermore, no data are yet available with respect to the specific radiosensitivity of the different cellular components of the immune system, or the microenvironment, and no data are available describing effects of radiation on circulating immune cells.

The here presented study describes a clinical phase I/II trial in pancreatic cancer patients with scheduled resection. The pancreatic cancer will be irradiated two days prior to planned surgery using precision external beam radiotherapy by photon intensity modulated radiotherapy (IMRT). Primary endpoints are the local radiation dose leading to tumor infiltrating T4 cells as a surrogate parameter for antitumor activity. Secondary objectives include clinical parameters such as local tumor control, patient survival, treatment related toxicity as well as quality of life of the patients. Furthermore, frequencies of tumor reactive T cells in blood and bone marrow will be correlated with blood cell whole genome transcriptional and plasma-protein analyses.

## Methods/Design

### Trial Organisation/Coordination

This trial is organised by the joint trial office of the Departments of Visceral Surgery and Radiation Oncology of the University Hospital Center and the Radiation Oncology Department of the German Cancer Research Center (dkfz), Heidelberg, Germany. The radiotherapy will be carried out by the Radiation Oncology at dkfz and surgery will be performed at the Surgery Department of the University Hospital Center Heidelberg. Translational studies will be carried out by the Department of Translational Immunology of dkfz, the Radiation Oncology Departments if dkfz and the Surgery Departments of the University Hospital. This trial is an investigator initiated trial and is coordinated by the Radiation Oncology Department of dkfz and the Department of Visceral Surgery of the University Hospital Center Heidelberg. The Radiation Oncology Department of dkfz is responsible for the overall trial management, trial registration, database management, quality assurance including monitoring and scientific program of all trial related meetings. **(Trial number NCT01027221)**

### Ethical and legal considerations

The final clinical trial protocol, the patient information and informed consent sheets were approved by the independent ethics committee of the University of Heidelberg S080/2008 (http://klinikum.uni-heidelberg.de). Further, the study was approved by the German Federal Authorities for Radiation Protection (Bundesamt fuer Strahlenschutz, Salzgitter, Trial Number Z5-22461/2-2009-003) Written informed consent is obtained from each patient in oral and written form before inclusion in the study. No data specifically required for this trial will be obtained if informed consent is not given. Patients are informed about the strict confidentiality of their personal data within this trial, but their pseudonymised medical records may be reviewed for trial purposes by authorized individuals other than their treating physician.

Patients will be informed about the additional specific risk of a bone marrow aspiration, which is voluntarily and will not lead to exclusion from the study in case of refusal.

It will be emphasized that the participation is voluntary and that the patient is allowed to refuse further participation in the protocol whenever he/she wants. This will not prejudice the patient's subsequent care. This study is carried out in accordance to the current Declaration of Helsinki (sixth revision, 2008), the principles of "Good Clinical Practice"; (GCP), and the Federal Data Protection Act. It is registered at the Clinical-Trials.gov protocol registration system (http://www.clinicaltrials.gov) and the assigned identification number is NCT01027221

### Study design

This single fraction radiation on operable pancreatic cancer trial is a registered (ClinicalTrials.gov - NCT01027221), investigator initiated, prospective, randomized controlled phase I/II trial meant to evaluate the optimal neoadjuvant, single fraction radiation dose for patients who will undergo resection for colorectal liver metastases in curative intent. Patients will be randomised either to the control group -receiving no radiation- or the three treatment groups - receiving 0.5 Gy, 2 Gy or 5 Gy single fraction radiation to the metastasis 48 h prior to the resection of the metastasis. The treatment is offered to a heterogeneous group of patient of both sexes, covering a wide range of comorbidities. For the irradiation a highly conformal dose distribution to the tumor with 2 cm safety margin is delivered using a 6 MV LINAC and IMRT treatment application.

After the inclusion of 20 patients an interims analysis will be performed. The final analysis will be performed within three month of the inclusion of the last patient. The trial design will not be changed without prior agreement of the ethics committee.

### Safety aspects and adverse events

Due to the relatively low dose of the applied radiation dose to the tumor only, there are only very few potential local adverse events to be expected (AE). Potential AE include skin alterations e.g. redness, transient nausea, diarrhea or temporary elevated transaminases, although these AEs even rarely occur in conventional radiotherapy using considerably higher doses (e.g. neoadjuvant 5 × 5Gy for rectal or gastric cancer, 54 Gy in pancreatic cancer). Possible AEs will be scored by the RTOG Scale.

### Objective of the study

The primary objectives of this trial are the determination of the single fraction radiation dose, which leads to an optimal anti-tumor immune response. The number of tumor infiltrating T-cells will serve as a surrogate marker for anti-tumor immune response. Secondary molecular objectives are the T cell activity in the resected tumor, the density of regulatory T cells in the resected tumor, the frequency of tumor reactive T cells in blood and bone marrow in correlation with changes in the level of proteins involved in immune response or angiogenesis and transcriptomics in whole blood cells. Further clinical secondary objectives are acute radiogenic toxicity, postoperative morbidity and mortality, local control, recurrence patterns, overall survival and quality of life.

### Trial population

This trial focuses on patients with resectable liver metastases of colorectal cancer. The study includes patients over 50 years of age. Based on the preoperative imaging the surgeon determines if the pancreatic tumor can be resected in curative intent. Patients with unresectable pancreatic cancers, metastasis, secondary malignancies, liver cirrhosis or previous radiotherapy to the upper abdomen will be excluded from the trial. A detailed overview of all eligibility and exclusion criteria is given in Table 1.

**Table 1:** Eligibility criteria

Inclusion criteria
Resectable pancreatic Carcinoma
Written informed consent of the patient
No evidence of active or former concurrent malignant diseases

**Exclusion criteria**

Previous radiotherapy to the upper abdomen
Participation in other therapeutical trials
Pregnancy
No willingness on regular follow up care

### Screening/Randomisation/Intervention

Patients will be screened by the Surgery Department for inclusion criteria. Patients are randomly allocated to either study intervention to balance treatment groups for all known and unknown potentially confounding factors. A block randomisation list is created via computer system. The sealed randomisation list is stored in the principal investigator's file. Patients are randomised using consecutively numbered opaque envelopes prepared and sealed by an independent study nurse. All patients screened, including those, who did not give informed consent, are entered in a consecutive list.

Given the consent, patients are randomized either to the control group - receiving no radiation - or the three irradiation treatment groups. Within the irradiation treatment groups patients will receive 0.5 Gy, 2 Gy or 5 Gy single fraction radiation 48 h prior to resection. After the adjustment of an individual positioning device, computed tomography and, if needed, magnetic resonance imaging (MRI) treatment planning examinations are performed. After 3D inverse treatment planning and plan optimizing using VIRTUOS and KONRAD software, intensity modulated radiation therapy will be administered using five to nine 6 MV (LINAC) photon beams. Immediately prior to the irradiation the setup error of the patient will be determined via In-Room-CT, and corrected if necessary.

Venous blood will be obtained (54 ml) on the day of radiation treatment planning. A second blood sample will be obtained just prior to the resection before anaesthesia. A bilateral bone marrow sample (each 27 ml) will be taken after induction of general anaesthesia immediately before the skin incision after disinfection from both iliac crests. Bone marrow sampling is optional for the patient. EDTA and not Heparin will be used as anticoagulant for bone marrow and blood samples. After the resection of the tumor a part of the tumor will be immediately removed and stored at -80°C for further T cell and other analyses.

On the postoperative day seven and at the follow-up/restaging visits (4 weeks postoperatively, 4 and 7 months postoperatively) additional venous blood samples (54 ml) will be obtained. Quality of Life will be tested using the EORTC QLQ C-30 and the additional PAN-26 at 4 months and 7 months postoperatively. Clinical examination will be performed and blood samples will be obtained by the investigators at all visits.

### Sample size and statistical consideration

Based on the study design, this monocentric randomized controlled trial is planned as a comparison of four parallel groups.

Group 1: Dose = 0 Gy (control)

Group 2: Dose = 0.5 Gy

Group 3: Dose = 2 Gy

Group 4: Dose = 5 Gy.

Aim of the study is to determine the maximum active radiation dose with three different doses. This will be carried out hierarchical, in two consecutive steps.

1. Step: Comparison of the three doses against the control group ("Many-to-one" comparison after Dunnet)

2. Step: Comparison of the three doses among each other ("all-subset" comparison) Each of the two steps happen on multiple level of significance of p < 0.05. The power of each step should be at least 80%.

Based on advanced information of a study by Galon et al. in colorectal cancer patients, the density of tumor infiltrating T cells is a relevant prognostic parameter. Patients with a good prognosis had a mean of x-good = 600 CD8 T cells/mm^2^, the group with the bad prognosis had a mean of x-bad = 370 CD8 T cells/mm^2^. The standard deviation in both groups was 50/mm^2^. Further analysis revealed that patients with x = 300 tumor infiltrating CD8 T-cells/mm^2 ^in tumor tissue had a significantly improved prognosis. Given this background, a clinical relevant difference between 100 and 200 CD8 T-cells/mm^2 ^seems plausible. The number of cases should be sufficient for a difference of 150 CD8 T-cells/mm^2^.

For the first step n = 32 analyzable patients will be needed, equals n = 8 for each group. The nominal power of ANOVA is around 90%. For the second step (comparison of the best group, Hsu 2006), the power for n = 32 is 95%. Not all of the randomized patients will be analyzable with respect to the primary objective. A failure of 10 - 15% has to be assumed due to bad tissue quality. Therefore n = 40 patients should be randomized.

The calculated number of cases was carried out with PASS 2005 (Hintze J, 2004): NCSS and PASS. Number Cruncher Statistical Systems. Kaysville, Utah. http://www.ncss.com) with the procedure for multiple comparisons by Jason C. Hsu (1996) (Multiple Comparisons: Theory and methods, Chapman & Hall). The analysis of the primary objective results confirmatory on a multiple level of significance of p < 0.05 by ANOVA based on the calculated number of cases. The analysis of the secondary objective results is descriptive by determining the means, the quartiles as location parameter and the standard deviation and range as dispersion parameters. The correlation between the parameters will be quantified by the Pearson or Spearman test.

### Study objectives and endpoints

The primary objective of this clinical trial is the amount of tumor infiltrating T cells, defined by the total number of CD 8 T cells. Secondary molecular objectives are the T cell activity in situ, the amount of regulatory T cells in situ and the frequency of tumor reactive T cells in blood and bone marrow of the patients. Furthermore, the study will analyze migration relevant adhesion molecules on tumor related vascular endothelial cells, the expression of proinflammatory cytokines, and immune-associated transcription factors in tumor and blood of the patients. Whole genome wide expression profiling in whole blood cells will be correlated with clinical outcome and with immunological response. Additional secondary clinical objectives are local and systemic morbidity

All secondary objectives are presented in Table 2.

**Table 2:** Secondary endpoints

• Local control and recurrence patterns of pancreatic cancer
• Progression free survival
• Radiation related toxicity
• Surgical morbidity in patients who received this protocol treatment
• 30 day postoperative mortality
• Quality of life according to EORTC QoL questionnaire after 3 and 6 months
• Plasma proteomics
• Whole blood transcriptomics

### Follow up

The patients will undergo a routine follow up (physical examination of the patients, tumor markers (CA 19-9), CT/MRI) every three months. Disease free survival and overall survival will be recorded. The clinical management and postoperative treatment of the patients will not be influenced by the results of this study. Decisions about further adjuvant treatment (e.g. chemotherapy) of the patients are left to the discretion of the treating oncologist.

### Data management and quality assurance

This trial is coordinated by the Radiation Oncology Department of the German Cancer Research Center. The data regarding T cell detection in blood and bone marrow of all patients will be entered in a password-protected database at the Department of Surgery, University of Heidelberg and at the German Cancer Research Center. The clinical data of all included patients will be centrally collected in a database located at the Department of Radiation Oncology at dkfz. The clinical and laboratory data will be merged in the password-protected database at the Department of Surgery, University of Heidelberg, Germany and at the German Cancer Research Center.

### Current status

At the current time of writing we have randomized 17 patients. The duration of the trial is expected to last 9 more months. A scheduled interims analysis will be performed following the inclusion of the first 20 patients. Evaluation and reporting of the clinical and laboratory results will be done within 3 months of the end of recruitment and closing of the database.

## Competing interests

The authors declare that they have no competing interests.

## Authors' contributions

CT participated in protocol design, ethical committee and BfS applications, trial coordination and conduction, patient recruitment, translational studies, and wrote the manuscript together with PEH. HSW participated in developing the protocol concept, participated in the ethical committee application, trial coordination and patient recruitment. FK carries out immuno monitoring and participates in trial coordination. FFFR participates in trial conduction. AB carries out immuno monitoring. CR participates in patient recruitment. NR participates in trial conduction. MK participates in trial conduction. ChrT participates in patient recruitment. MWB participates in trial conduction. JD participates in trial conduction. JW participates in trial conduction. PB participated in developing the study concept, participates in trial conduction and coordinates the immuno monitoring. JW participated in developing the study concept, and participates in trial coordination and conduction. PEH coordinated the study concept and protocol design, led the approval committee applications, coordinates the trial conduction and translational studies, and wrote the manuscript together with CT. All authors read and approved of the final manuscript

## Acknowledgements

This work is supported in part by grants from Nationales Centrum fuer Tumorerkrankungen (NCT)/Tumorzentrum Heidelberg and Kompetenzverbund Strahlenforschung (KVSF, 03NUK004A,C) of Bundesministerien fuer Bildung,  Forschung und Umwelt (BMBF/BMU).

## Pre-publication history

The pre-publication history for this paper can be accessed here:

http://www.biomedcentral.com/1471-2407/11/134/prepub
